# Predictors of Lesions Contiguity and Transmurality in Canine Ventricular Models After Catheter Ablation

**DOI:** 10.3389/fcvm.2022.920539

**Published:** 2022-06-23

**Authors:** Abdel Hadi El Hajjar, Mario Mekhael, Chao Huang, Charbel Noujaim, Yichi Zhang, Eugene Kholmovski, Tarek Ayoub, Chan Ho Lim, Nassir Marrouche

**Affiliations:** ^1^Department of Cardiology, Tulane Research Innovation for Arrhythmia Discoveries, Tulane University School of Medicine, New Orleans, LA, United States; ^2^Department of Biomedical Engineering, Johns Hopkins University, Baltimore, MD, United States

**Keywords:** ventricular arrhythmia, catheter ablation, transmurality, interlesion distance, contiguous lesions

## Abstract

**Background:**

Interlesion gaps and transmurality of lesions after catheter ablation can precipitate suboptimal efficacy in preventing arrhythmias.

**Aims:**

We aim to assess predictors of acute transmural lesion formation and the interlesion distance threshold for creating a continuous, chronic scar after ventricular ablation.

**Materials and Methods:**

Ablation procedures were performed on 7 canines followed by late gadolinium enhancement MRI (LGE-MRI). Transmurality of lesions was assessed by 2 independent operators. Ablation parameters such as duration (s), power (W), temperature (C), contact force (CF) (g), were collected for each ablation point. After 7-12 weeks, LGE-MRI was performed, followed by euthanasia, and heart excision. Some lesions were created in pair. Lesion pairs were spaced 7-21 mm apart as measured by Electroanatomic mapping (EAM), with different operating parameters (power 35 or 50W, duration of energy delivery 10, 20 or 30s and contact force of 10g or above). We performed a logistic regression analysis to determine predictors of transmural lesion formation.

**Results:**

Eighty-one radiofrequency ablation were performed in total [33 in the Left ventricle (LV) and 48 in the Right ventricle (RV)]. Higher CF was a significant predictor of transmural lesion formation (β = 0.15, OR = 1.16, 95% CI [1.03 – 1.3], *p* = 0.01), and lesions delivered in the RV were more frequently transmural than lesions delivered in the LV (β = −2.43, OR = 0.09, 95%CI [0.02 – 0.34], *p* < 0.001). For the paired analysis, thirty-eight lesions were created contiguously: fourteen connected lesions and twenty-four unconnected lesions. EAM distance was significantly larger in unconnected lesions than connected lesions (16.17 ± 0.92 mm vs. 11.51 ± 0.68 mm, respectively, *p* < 0.05). We concluded that an interlesion distance of less than 10 mm is required to prevent gap formation. Average volumes in unconnected lesions (*n* = 24) at the acute and chronic stages were 0.55 ± 0.11 cm^3^ and 0.20 ± 0.02 cm^3^, respectively. On average, lesion volumes were 64% (*p* < 0.05) smaller at the chronic stage compared to the acute stage. Among connected lesions (*n* = 14), we observed a volume of 1.19 ± 0.8 cm^3^ and 0.39 ± 0.15 cm^3^ at the acute and chronic stages, respectively. These connected lesions reduced in volume by 67% on average.

**Conclusion:**

To create contiguous scars on the ventricular endocardial surface, paired lesions should be spaced less than ten millimeters apart. Higher contact force should be used in ventricular ablation to create transmural lesions.

## What’s New?

- Paired lesions created in the ventricles should be spaced less than ten millimeters apart to ensure continuity and prevent gap formation.
- Chronic scar obtained after catheter ablation can be accurately assessed by LGE-MRI, when compared to histological assessment.
- Higher contact force should be used in ventricular ablation to create transmural lesions.

## Introduction

Radiofrequency ablation (RFA) can treat cardiac arrhythmias. Pulmonary vein isolation (PVI), used for treating Atrial Fibrillation (AF), is achieved by a series of contiguous lesions created by RFA. But RFA can also be used in ventricular arrhythmias.

The outcome of RFA ablation relies on many parameters, such as contact force, power, duration, and impedance. The Ablation index (AI) incorporates force, power and duration in a weighted formula to predict lesion size and depth (1). Most labs use the following ablation parameters: a minimum contact force of 5–10g, delivering a power of 25–35W for a duration of 20–60 s. Recent studies are focusing on the benefits of a “high power (40–50W)-low duration” ablation protocol (2). However, while “high power- low duration” RFA resulted in similar lesion volumes compared to “low power- long duration” RFA, the former also produced shallower lesion depth (3). The lesion depth and diameter increased significantly with greater contact forces (2).

Compared to other well-studied ablation parameters, there is currently a knowledge gap regarding lesion durability and the required distance between two lesions (“interlesion distance”). Also, transmurality of ablation was also shown to be an important determinant of treatment success (4–7). Ensuring the durability and transmurality of lesions created with RFA is critical as any electrical reconnection can lead to recurrence of the arrhythmia (8). It is also imperative to minimize interlesion distance, as gap formation after ablation can also lead to arrhythmia recurrence.

Thus, the main goal of our study is to assess predictors of acute transmural lesion formation and the optimal interlesion distance in ventricular ablation that will create durable, connected lesions, using canine models.

## Materials and Methods

### Study Goals

Here, we investigate the interlesion distance threshold at which paired lesions become connected. Also, we want to assess the predictors of acute transmural lesion formation in the ventricles.

### Animal Preparation

The experimental protocol conforms to the Guide for the Care and Use of Laboratory Animals. The Institutional Animal Care and Use Committee at the University of Utah approved the study. The study was conducted on seven adult (25–30 kg), male mongrel dogs and Class A purpose-bred for biomedical research. All MRI and ablation procedures were performed with the animals under anesthesia. Induction of anesthesia was performed through intravenous injection of propofol (0.1–8 mg/kg). Deep anesthesia was maintained with isoflurane (1.5–3.5%) under mechanical ventilation.

### Timeline of Procedures

Initially, animals were scanned via MRI to establish the baseline anatomy of the cardiac chambers. Approximately one week later, the first of two ablation procedures were performed followed immediately by MRI of these acute lesions. Between 7 and 12 weeks following the first ablation, a terminal study was performed whereby an additional ablation procedure was carried out which was immediately followed by MRI, euthanasia, and heart excision.

### Radiofrequency Ablation and Electroanatomic Mapping

Anesthetized animals were moved to the electrophysiology (EP) laboratory and set up with a fluoroscopy (Artis Zeego; Siemens United States, Malvern, PA) and a CARTO electroanatomic mapping system (Biosense Webster, Diamond Bar, CA). We initially gained access to the right femoral vein via percutaneous puncture with ultrasound-guidance. An 11F sheath was placed in the vein through which a SoundStar 3D Ultrasound Catheter (Biosense Webster) was advanced into the right atrium. An atrial transeptal puncture was obtained, and an irrigated Thermocool SmartTouch SF ablation catheter (Biosense Weber) was advanced into the left atrium. Some ablations lesions were made in pairs within the left ventricle, and right ventricle. Lesions within each pair were spaced 7–21 mm apart as measured by EAM. In other terms, interlesion distance was measured as the distance between the tip of each catheter used to create the ablation lesion on the endocardial surface. For the paired lesions, ablation was performed using a range of the following operating parameters: Power (35 and 50W), duration of energy delivery (10, 20, and 30s), and contact force (> 10g). Temperatures were similar to temperatures used in clinical settings and all temperatures for each ablation point were collected. Three subsets of lesions were created: 35W-30s, 50W-20s and 50W-10s.

### Magnetic Resonance Imaging of Ablation Lesions

#### Baseline Magnetic Resonance Imaging Studies

All baseline MRI studies were performed on 3 Tesla MRI scanners (Trio or Prisma; Siemens Healthcare, Erlangen, Germany) using body and spine phased-array coils. Baseline imaging protocol included T1 and T2 mapping, double inversion recovery (DIR) prepared T2-weighted (T2w) turbo-spin echo (TSE) sequence, 3D T1-weighted (T1w) sequence, contrast-enhanced MR angiography (Gd-BOPTA, 0.15 mmol/kg, Bracco Diagnostic Inc., Princeton, NJ) and post-contrast 3D T1w and 3D late gadolinium enhancement (LGE) sequences. LGE scans were repeated at different time points after contrast injection. The main goals of baseline MRI study were to acquire images for CARTO system and to obtain pre-ablation images for ablation injury detection. Measurements of acute and chronic lesions included all changes due to the ablation point such as edema and/or scar formed.

#### Acute Magnetic Resonance Imaging Studies

At the end of the ablation procedure in the EP suite, each animal was moved to the MRI suite. The time lapse between the end of ablation procedure in the EP suite and the animal in the MRI suite was less than an hour. MR imaging technique and parameters used were similar to that of the baseline and acute MRI. The main goal of acute MRI study was to visualize various components of ablations [edema, micro-vasculature obstruction (MVO), and lesion core]. Also, the classification of connected versus unconnected lesions were defined on acute MRI studies.

#### Terminal Magnetic Resonance Imaging Studies

Two months after the initial ablation, the MRI study was repeated. MR imaging technique and parameters used were similar to that of the baseline MRI. The main goals of terminal MRI study were to image the state of the chronic lesions approximately 2 months after the initial ablation procedure, as well as to re-evaluate various components of acute ablations [edema, micro-vasculature obstruction (MVO), lesion core]. At the end of this study, animal was euthanized, and the heart extracted for *ex vivo* high-resolution (isotropic 0.5 mm) MRI, as well as macroscopic and histological examination.

#### Scan Parameters

The parameters for the different MRI scans were as follows. Non-contrast T1w - respiratory navigated, ECG triggered, saturation recovery (SR) prepared turbo-FLASH sequence with repetition time (TR) = 3.1 ms, echo time (TE) = 1.4 ms, flip angle (FA) = 12o, inversion time (TI) = 400 ms, voxel size = 1.25 × 1.25 × 2.5 mm, fat saturation. DIR T2w TSE - respiratory navigated, ECG triggered, DIR prepared 2D TSE pulse sequence with TE = 81 ms, TR = 3 cardiac cycles, echo train length = 21, fat suppression using SPAIR, in-plane resolution of 1.25 × 1.25 mm, slice thickness of 4 mm. Contrast-enhanced MRA - respiratory navigated, ECG triggered, SR prepared 3D turbo-FLASH with resolution = 1.25 × 1.25 × 2.5 mm, TR/TE = 2.9/1.3 ms, FA = 17o, TI = 120 ms, fat saturation. Post-contrast T1w - respiratory navigated, ECG triggered, SR prepared 3D turbo-FLASH with resolution = 1.25 × 1.25 × 2.5 mm, TR/TE = 3.1/1.4 ms, FA = 15o, and TI = 150 ms. LGE - respiratory navigated, ECG triggered, inversion recovery (IR) prepared 3D turbo-FLASH with resolution = 1.25 × 1.25 × 2.5 mm, TR/TE = 3.1/1.4 ms, flip angle = 14o, TI = 230 = 330 ms, fat saturation.

### Pathological Assessment of Lesions

The heart was fixed in formaldehyde. Then, it was cut in 2 to 3 mm thick slices that were photographed on both sides in high resolution. Tissue was stained using Masson Trichrome staining protocol for better lesion visualization. The area of the lesion (average of the calculated area on the two sides of the slice) was multiplied with the slice thickness to estimate the lesion volume for each slice. The volume across all the slices for each lesion was added to get the total lesion volume. For pathology/histology comparison, depth and width were measured in the slice with higher depth and width.

### Analysis of Transmurality

Transmurality of lesions was assessed by 2 independent operators. Each operator reviewed all sections obtained from LGE-MRI and lesion depth was assessed as the amount of scarred tissue between the endocardial and epicardial surfaces. Transmurality was considered as a dichotomic variable: transmural meaning lesions occupied the entire thickness of the wall from the endocardial to the epicardial surface; non-transmural, meaning lesions did not occupy the entire thickness of the wall. The operators reviewed individually each voxel of the wall in areas occupied by lesions.

Lesions were then assessed pathologically after euthanasia and heart excision. A lesion was considered transmural on pathology if the scarred tissue occupied the entire region of the wall.

Ablation parameters such as duration (s), power (W), temperature (C), contact force (CF) (g), were collected for each ablation point. We performed a logistic regression analysis to determine predictors of transmural lesion formation. Transmurality was considered a dependent variable and duration, power, temperature, contiguity of lesions, chamber [LV (+) or RV (−)] were considered as independent variables.

### Analysis of Lesion Volumes and Distances Between Paired Lesions

Segmentation of ventricular walls and ablation lesions from LGE-MRI scans were performed with custom software (Corview, Marreck Inc., Salt Lake City, UT) using the fast growcut algorithm (9). In short, we defined gross boundaries of a lesion to be segmented by drawing seed and background pixels in and around it. The fast growcut algorithm propagates these input labels based on principles derived from cellular automata to classify all the pixels. Refinements of the segmentation can be inputted in the form of additional seeds and background pixels by the user until the lesion is sufficiently segmented. Using this tool, volumes of segmented lesions at the acute and chronic stage were measured. The center-to-center and gap distances between pairs of segmented lesions were also measured using the bwconncomp and bwperim functions in MATLAB (The MathWorks Inc, Natick, MA).

### Definition of Connected and Unconnected Lesions

Lesions were classified as connected or unconnected based on LGE-MRI. Two lesions were considered connected if the edge of one lesion is not well defined and intersects with the edge of the other lesion. In other terms, two lesions were considered connected if no gap was present between two contiguous lesions on LGE-MRI. If a gap is present or the edges/borders of the two lesions are well defined, lesions were considered unconnected. Only for unconnected lesions, edge-to-edge, and center-to-center distances between two paired lesions were measured using the Corview Software. Edge-to-edge distance was measured where the two lesions were closest to each other. Those measure were not calculated for connected lesions as edge-to-edge distances are equal to 0 and borders are not well defined to determine lesions’ centers.

### Statistical Analysis

Statistical data are presented as mean ± standard error.

First, to assess for predictors of transmurality, we used a backward stepwise logistic regression model with the following parameters: duration (s), power (W), temperature (C), contact force (CF) (g), chamber (odds that lesions are transmural in the LV over the odds of lesions being transmural in the RV) and whether lesions were connected to other lesions (contiguity) or not. Second, the lesions were divided into connected and unconnected lesions. To compare means of continuous variables (Force, Temperature, FTI, Impendence drop), between the two groups groups, *t*-test or Mann Whitney test were performed depending on normality of the distribution. Normality was assessed using Shapiro-Wilk test. Third, *t*-test was used to assess lesion depth and width differences between histology and LGE-MRI as distributions were normal. We also included Bland Altman Plots to compare LGE-MRI and pathological assessment of lesions.

Finally, EAM distance between paired connected lesions and unconnected lesions were performed using *t*-test as distributions in respective groups were normal. All statistical analyses were performed using SPSS (version 27.0).

## Results

### Summary of Lesions Characteristics

We created 81 radiofrequency ablation lesions in the left (LV) and right (RV) ventricles of 7 canines. Out of those 81 lesions, 33 ablation lesions were in the LV and 48 in the RV.

Out of the 81 lesions, we performed thirty-eight paired ventricular lesions. A total of twenty-four unconnected (twelve pairs) lesions were created and equally distributed in the left ventricle (LV), right ventricle (RV) and septum. A total of fourteen connected lesions were created (seven pairs), eight of which are in the LV. There was no significant difference in force and temperature used in both groups (*p* > 0.05). However, there was a significant difference in EAM distance between connected and unconnected lesions (*p* < 0.05).

**FIGURE 1:**
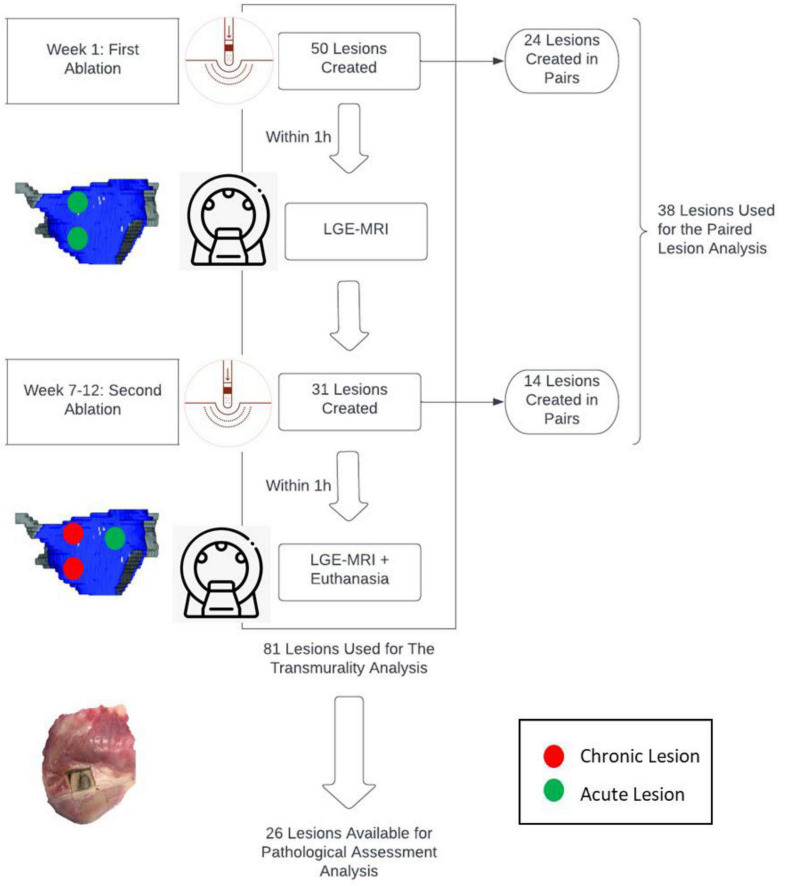
Flow Chart detailing the lesions created in canine models.

All characteristics of the paired lesions are summarized in Table 1. A flow chart summarizing the study is seen in Figure 1.

**TABLE 1:** Characteristics of connected and unconnected lesions.

	Unconnected lesions	Connected lesions	*P*-value
**Location**			
LV free wall*	8 (33.3%)	8 (57.1%)	–
RV free wall*	8 (33.3%)	2 (14.3%)	–
Septum*	8 (33.3%)	4 (28.6%)	–
**Ablation parameters**			
35W-30 S*	4 (16.7%)	10 (71.4%)	–
50W-10 S*	2 (8.3%)	2 (14.3%)	–
50W-20 S*	18 (75%)	2 (14.3%)	–
Force average (in grams)*	13.5 (7.9–19.1)	15 (11.9–18.1)	0.183
Temperature average (in°Celsius)*	33.4 (28.2–38.6)	32.1 (28.8–35.38)	0.217
EAM distance average (in mm)	16.2 (13.8–18.6)	11.5 (9.1–13.9)	< 0.001
Force time integral (FTI)	340.8 (172.8–508.8)	392.6 (230.6–554.6)	0.405
Impedance drop (in ohm)	36.14 (24.84–47.44)	32.54 (17.39–47.69)	0.432

LV: Left Ventricle, RV: Right Ventricle, EAM: Electroanatomic Mapping, W: Watts, S: Seconds, Mm: Millimeters. *Results are reported in numbers (percentage); Other reports are reported as mean (± standard deviation).

### Transmurality Analysis

a- **Predictors of acute transmurality of lesions as defined by LGE-MRI**

**FIGURE 2:**
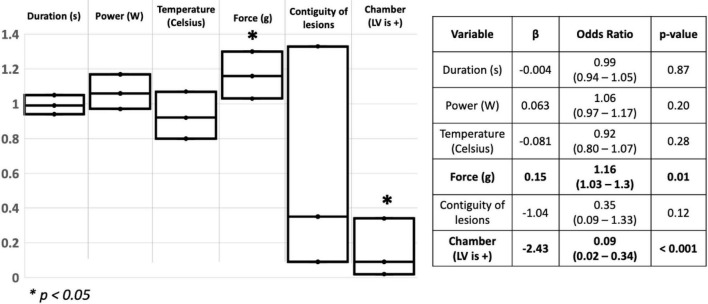
Schematic representation of the logistic regression used to assess for predictors of transmurality in the ventricles. *S: Seconds; W: watts; g: Gram; LV: Left ventricle*. *p < 0.05.

Out of the 81 lesions created, we delivered 33 ablation lesions in the LV and 48 in the RV. Of the lesions delivered in the LV, 4 were transmural while 23 were transmural in the RV. We found that higher CF was a significant predictor of transmural lesion formation (β = 0.15, OR = 1.16, 95% CI [1.03 – 1.3], *p* = 0.01), and lesions delivered in the RV were more frequently transmural than lesions delivered in the LV (β = −2.43, OR = 0.09, 95%CI [0.02 – 0.34], *p* < 0.001) (Model fit: Chi-Square = 26.75, *p* < 0.001). Other ablation parameters were not significant predictors of transmural lesions in our cohort. Results of the logistic regression are shown in Figure 2.

b- **Data on transmurality after histological assessment**

**FIGURE 3:**
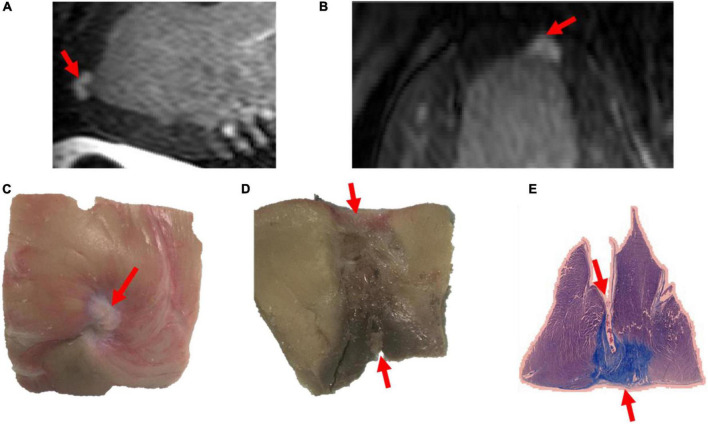
Images of a transmural lesion in the LV free wall on LGE-MRI [**(A)**: sagittal view; **(B)**: axial view] and pathology [**(C)**: epicardial view; **(D)**: axial view; **(E)**: view on microscopy].

A total of 26 lesions were available for pathological assessment. Lesions were assessed pathologically after euthanasia for width, depth and transmurality. In terms of transmurality, lesions seen on LGE-MRI were very similar to lesions assessed on histology. Four lesions showed transmurality on LGE-MRI and 22 lesions were non-transmural. After pathological assessment by an operator blinded to LGE-MRI results, results were similar to those obtained on LGE-MRI: four lesions were transmural and 22 were not. Amongst the transmural lesions, two lesions were created in the RV free wall using 35W-30S and the two others were created in the LV apex using 50W-20S. Images of a transmural lesion are shown in Figure 3.

Out of the lesions available for pathological assessment, 15 were chronic lesions and 11 were acute lesions. In the acute lesions’ subset, 10 lesions were non-transmural on both LGE-MRI and histology and one lesion was transmural in both LGE-MRI and histology. Out of the chronic lesions’ subset as seen on LGE-MRI, 12 lesions were non-transmural both in the acute and chronic settings, and 3 were transmural both in the acute and chronic settings. On histology, 12 lesions were non-transmural, and 3 lesions were transmural.

c- **Comparison between LGE-MRI lesions and pathological assessment post necropsy**

**FIGURE 4:**
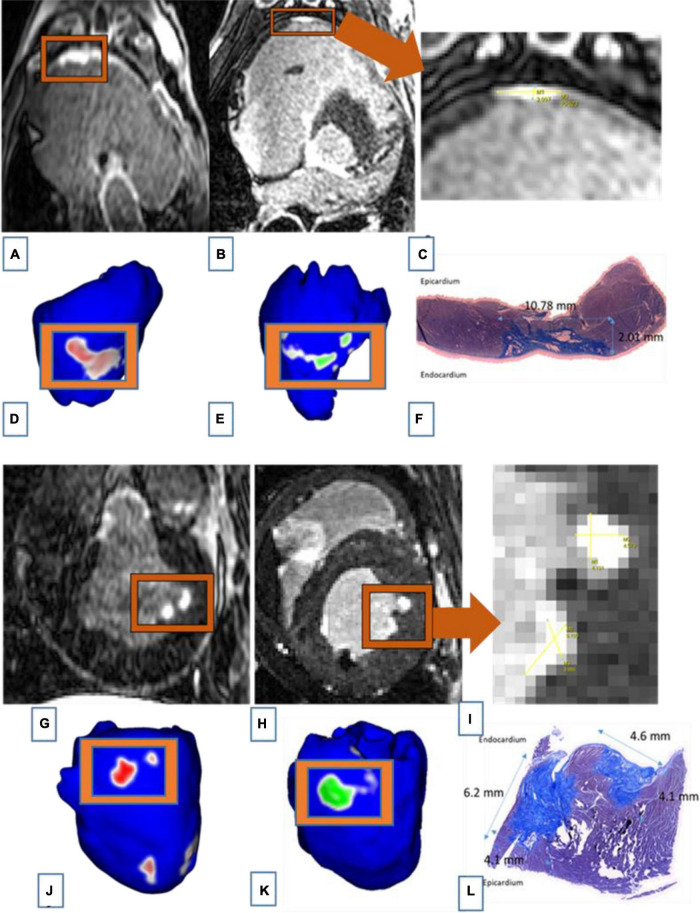
Images of two connected lesions in the RV free wall shown on LGE-MRI and corresponding Corview reconstructions in the acute phase [**(A):** axial view and **(D)**] and the chronic phase [**(B)**: axial view and **(E)**]. Note the similarities between LGE-MRI in the chronic settings **(B)** and pathological assessment **(F)**. Images of two unconnected lesions in the LV free wall shown on LGE-MRI and corresponding Corview reconstructions in the acute phase [**(G):** axial view and **(J)**] and the chronic phase [**(H)**: axial view and **(K)**]. Note the similarities between LGE-MRI in the chronic settings **(H)** and pathological assessment **(L)**. Images **(C)** and **(I)** are zoomed in figures of B and H, respectively.

The similarity between LGE-MRI and pathological assessment can be seen in Figure 4. Maximal lesion depth and width seen on histology as compared to LGE-MRI are summarized in Table 2. There was no statistically significant difference between histological findings and LGE-MRI measurements (*p* = 0.973 for width; *p* = 0.741 for depth).

**TABLE 2:** Comparison of acute and chronic lesion measurements obtained using LGE-MRI and histological assessment.

	Histology	LGE MRI	*P*-value
**Lesion width (in mm)**			
Mean	6.213	6.22	–
Standard deviation	2.414	1.664	–
Standard error mean	0.670	0.462	–
Paired difference mean	−0.010 [95% CI: (−0.607, 0.588)]		0.973
Paired difference standard deviation	0.989		
Paired difference standard error mean	0.274		
**Lesion depth (in mm)**			
Mean	4.95	5.02	–
Standard deviation	2.174	1.585	–
Standard error mean	0.628	0.458	–
Paired difference mean	−0.075 [95% CI: (−0.564, 0.414)]		0.741
Paired difference standard deviation	0.769		
Paired difference standard error mean	0.222		

*Lesion width and depth are in mm; LGE-MRI: late gadolinium enhancement magnetic resonance imaging.*

We also included Bland Altman Plots as a Supplementary Figure 1 to compare LGE-MRI and pathological assessment of lesions.

### Paired Lesion Analysis

a- **Measured Lesion Volumes from LGE-MRI Reconstructions:**

• Unconnected Lesions

The average volumes of these lesions (2n = 24) at the acute and chronic stages were 0.55 ± 0.11 cm^3^ and 0.20 ± 0.02 cm^3^, respectively (2n = one pair of two lesions). Lesion volumes were on average 64% smaller (*p* < 0.05) at the chronic stage compared to the acute stage.

For the lesions created using 50W-20S, lesions reduced in volume on average from 0.52 to 0.21 cm^3^. For the two pairs created using 35W-30S, the average volume reduced from 0.75 to 0.15. For the paired created using 50W-10S, the volume reduced from 0.35 to 0.16.

• Connected Lesions

In these lesions, we observed a volume of 1.19 ± 0.8 cm^3^ and 0.39 ± 0.15 cm^3^ at the acute and chronic stages, respectively. These connected lesions reduced in volume by about 67%. The difference in volumes at the acute and chronic stages was statistically significant (*p* < 0.05).

For the lesions created using 35W-30S, lesions reduced in volume on average from 1.09 to 0.49 cm^3^. For the pair created using 50W-10S, the volume reduced from 0.3 to 0.24. For the pair created using 50W-20S, the volume reduced from 2.61 to 0.12.

Average volumes of all lesions created in the acute and chronic stages are shown in the Supplementary Figure 2. A detailed list of lesions created in pairs with ablation parameters are shown in the Supplementary Table.

b- **Distances Measured by EAM in Connected and Unconnected Lesions**

We compared the distance across the endocardial surface between various paired lesions as measured by EAM. Connected paired lesions had an interlesion distance of 11.03 ± 0.92 mm. Unconnected paired lesions had an interlesion distance of 16.17 ± 0.68 mm (*p* < 0.001).

**FIGURE 5:**
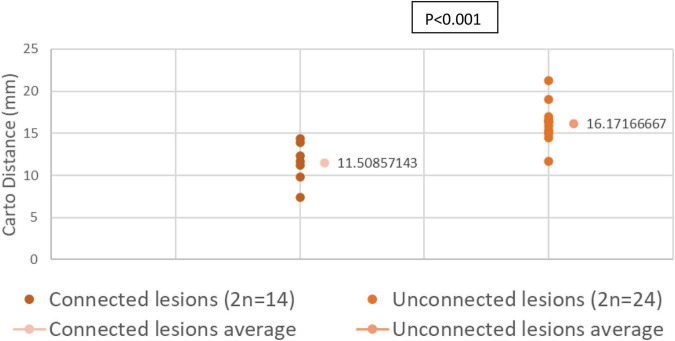
Distances across endocardial surface between various paired lesions as measured by EAM.

Distribution of interlesion distance in both connected and unconnected lesions are shown in Figure 5.

c- **Evolution of Connected and Unconnected Lesions with Time:**

All 24 unconnected lesions remained unconnected in the chronic settings. Out of the 14 connected lesions, one pair (2 lesions) became unconnected in the chronic settings, with an edge-to-edge distance of 1.3 mm. Interlesion distance on EAM used for this pair was 13.9 mm.

d- **Distances Measured on LGE-MRI in Unconnected Lesions:**

The average center-to-center distance between unconnected lesions as quantified by LGE-MRI was 24.27 ± 1.60 mm and 23.15 ± 2.91 mm at the acute and chronic stages, respectively. In addition, the edge-to-edge distance between these paired lesions as quantified by LGE-MRI at the acute and chronic stages were 4.35 ± 1.27 mm and 4.15 ± 0.95 mm, respectively. Center-to-center and edge-to-edge distances were reported on a limited number of paired lesions where clear lesion contours allowed calculation of such distances: Therefore, performing statistical analyses on these corresponding pairs was not possible due to the very limited number of pairs. No p-values were calculated.

**FIGURE 6:**
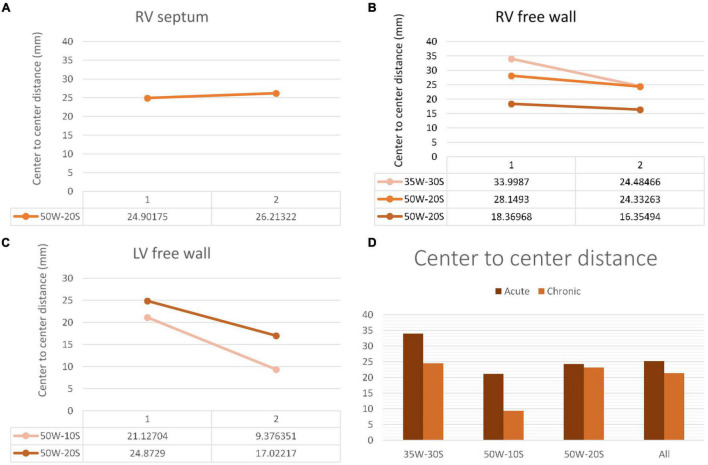
Center-to-center distance progression at varying doses and regions within the heart determined by MRI. The variation in center-to-center distance between the acute and chronic settings is shown in the RV septum **(A)**, RV free wall **(B)** and LV free wall **(C)**. Overall average of acute and chronic center-to-center distance between lesions created at varying ablation parameters **(D)**. Every line in each graph represents the variation of center-to-center distance between one pair of ablation points (50W-20S are shown twice as two pairs were created using this parameter). For instance, in the LV free wall, the center-to-center distance reduced from 21 to 9.3 mm from the acute to chronic settings in a pair of lesions. Another pair reduced from 24.9 to 17 mm.

**FIGURE 7:**
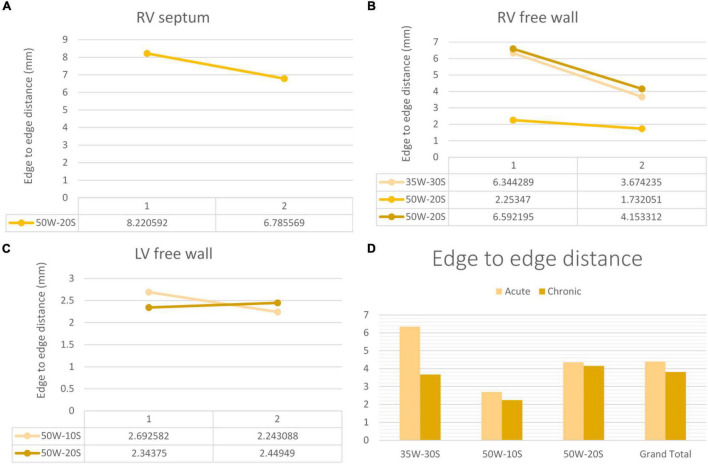
Edge-to-edge distance progression at varying doses and regions within the heart determined by MRI. The variation in edge-to-edge distance between the acute and chronic settings is shown in the RV septum **(A)**, RV free wall **(B)** and LV free wall **(C)**. Overall average of acute and chronic edge-to-edge distance between lesions created at varying ablation parameters **(D)**. Every line in each graph represents the variation of edge-to-edge distance between one pair of ablation points (50W-20S are shown twice as two pairs were created using this parameter) For instance, in the RV septum in panel **(A)**, the edge-to-edge distance reduced from 8.2 to 6.8 mm from the acute to chronic settings in a pair of lesions.

Edge to edge distance and center-to-center distances are shown in Figures 6, 7, respectively. According to our findings, lesions created in RV free wall and septum have little to no reduction in center-to-center distance. Instead, they demonstrate a small decrease in their edge-to-edge distance. However, lesions created in the LV tended to have a larger reduction in center-to-center distance with no change in their edge-to edge-distance.

## Discussion

In this study, we showed that the average endocardial distance between two ventricular lesions needs to be less than or equal to 10 mm for the pair to remain connected. Moreover, the minimum distance for two acute ablation lesions to be unconnected was 11.68 mm. Thus, clinicians should ensure an interlesion distance of < 10 mm while performing ablation procedure to safely achieve a continuous uninterrupted ablation line and scar. Finally, high contact force is required to create transmural lesions.

### Interlesion Distance in the Ventricles

A large interlesion distance may lead to the formation of gaps between lesions, which would result in a non-contiguous or non-transmural ablation scar (7). However, available data on ventricular ablation and gap formation are limited. Our study suggested that in two groups where force, force time integral, impedance drop, and temperature are similar, only interlesion distance was different between connected and unconnected lesions.

Scar-based ventricular tachycardia (VT) uses different strategies such as linear lesions in mapped sites and core lesion isolation (10). Tools to create contiguous lesions might optimize ablation strategies and reduce radiofrequency time. During core isolation in ventricular arrhythmias, we predict that having connected lesions is associated with less arrhythmia recurrence. Our rationale is that isolation of the ventricular scar is essential to prevent arrhythmia conduction. In fact, Ranjan et al. showed that in the ventricles, acute gaps that were non-conductive may become conductive when tissue is healed (11). This supports our hypothesis that minimizing gaps in ventricular ablations helps prevent arrhythmia recurrences. However, future studies need to address gaps in ventricular lesions. In terms of the potential applicability in the clinical setting, the findings of this study could one day be used to guide the procedure of core isolation of ventricular scar.

Our 10 mm threshold in the ventricles is larger than expected when correlated to clinical practices (12). Also, in our analysis, we focused on continuous, uninterrupted ablation lesion delivery, which is not always the case in clinical practice where sometimes ablation lesion may be interrupted. Recent evidence demonstrated the need for closer spacing of lesions if energy delivery was interrupted during ablation. In fact, lesions obtained with interrupted ablation resulted in smaller lesion volume (due to smaller width) compared to those achieved by continuous ablation (13). Ablation in human patients is more frequently interrupted than in animal studies. Also, ventricular wall is thick, thus allowing more space for propagation of radiofrequency heat wave. This may help explain why atrial lesions can require a lower interlesion distance threshold to achieve contiguity. Further investigations are warranted to explore the locational dependence of optimal interlesion distance in catheter ablation.

Note that all our ablations were performed using the Thermocool SmartTouch SF ablation catheter (Biosense Weber). EAM distances were calculated using the CARTO mapping system. Future studies should evaluate our findings using different catheters and other mapping systems.

### Lesion Size Regression

Our investigation is the first to study lesion evolution by looking at center-to-center and edge-to-edge distances. In the septum and RV free wall, edge-to-edge distances became smaller with time, but center-to-center distances stayed relatively constant. This can be explained by the time dependent resolution of edema and shrinkage of the lesion. However, in the LV free wall, even center-to-center distance became smaller. This suggests a greater degree of edema in the LV free wall compared to other locations. A possible explanation to this finding is that higher strain and contractile forces in the LV could lead to higher degrees of edema (14).

Our study also found that chronic lesion volumes were significantly smaller than acute lesion volumes. In unconnected lesions, chronic lesions were 64% smaller than their respective acute lesions. Understanding chronic lesion volume is crucial in preventing arrhythmia recurrence. In fact, it was shown that patients with minimal scar formation at 3 months after ablation (13% of LA myocardial volume enhancement on LGE-MRI) experienced higher recurrence rate of atrial fibrillation. In contrast, patients with moderate scar formation at 3 months had very high procedural success and a lower recurrence rate of atrial fibrillation (15). This decrease in lesion size is mainly explained by the subsiding edema and inflammation over time. Edema formation can be minimized by increasing contact force (> 12.5 g according to latest literature) (14). This phenomenon helps explain the dynamic evolution of the scar and can be monitored during catheter ablation procedure.

### Transmurality of Lesions

Transmurality of lesions created during ablation is a major predictor of ablation success (6, 7). Several studies have shown that some parameters might help predict transmurality (16, 17). For example, Squara et al. showed that contact force and force time integral are major components in transmurality formation (16). Our findings are similar to previous ones indicating that contact force is indeed the major ablation parameter involved in transmurality. However, unlike previous studies, our study had pathological validation of the lesions, thus giving more validation and relevance to those findings. In addition, our study showed that the most important predictor of transmurality was the location of the lesions, as RV lesions were significantly more transmural than LV lesions. Indeed, transmural lesion is easier to achieve on a thinner wall and therefore achieve better treatment success.

### Magnetic Resonance Imaging-Histology Lesion Size Comparison

There was a strong correlation between lesions seen on LGE-MRI and lesions assessed by histology as shown in Figures 3, 4. We have shown that ventricular ablation lesion size defined by MRI can be reliably associated with lesion size demonstrated by histological analysis (18). Dickfield et al. used a canine model to show that both T1-weighted (T1w) and T2-weighted (T2w) images were reliable in defining right ventricular ablation lesion size as seen on histology 12 h after the procedure. Though the T2w images were more consistent and showed higher signal intensity for the central necrosis zone compared to their T1w counterparts (19). Furthermore, the studies of Breen et al. (20) and Kholmovski et al. (21) also support the dependability of MRI in defining ablation lesion size as validated by histological specimen analysis.

Using a porcine model, Harrison et al. (22) demonstrated that T2w images were accurate at predicting acute atrial lesion sizes but often overestimated chronic lesion volumes, as validated by histopathological sections. Ranjan et al. (23) also used a porcine model to show that LGE-MRI can help acutely identify gaps in ablation lesions, which was verified by pathology analysis of the atrial tissue. Future techniques need to be developed to precisely assess reliability of MRI in the acute settings. In fact, acute edema might be underestimated because of its resorption when sampling the tissue.

### Study Limitation

First, the length between the acute and chronic MRI was variable in different animals (7 to 12 weeks). However, we do not expect a significant difference in scar maturation between 5 and 7 weeks. Secondly, our study focused on LGE-MRI only in terms of lesion analysis. Previous studies reported that native T1 MRI might be a better predictor of acute lesion size than LGE-MRI (21). However, we are studying the evolution of lesions at two timepoints with the same imaging technique. Native T1 does not accurately assess chronic lesions. Thirdly, three different ablation parameters were used (35W-30s, 50W-20s, and 50W-10s) introducing confounding bias in distance measurement. Unfortunately, this is a single-center, small sample study which initial goal was not the analysis of a single ablation parameter set. It is meant to be observational. Future studies should focus on comparing connected and unconnected lesions in a single parameter set and then dividing a larger sample into different sets. In addition, paired lesion analysis and transmurality analysis were performed separately in this study. However, both elements are crucial for ablation success. Thus, future studies with a larger number of lesions should be performed to assess predictors of both transmural and connected lesions. Finally, ablation energy applications in this study were heterogeneous and lesion numbers were small as only four lesions were created using the 50W-10s parameters. However, most ablations were performed using clinically relevant values. In fact, 50W-10s is not usually used in the clinical settings. Also, our study is meant to be observational. Future studies should include more variation in ablation parameters and performed on a larger scale to validate our results on predictors of transmurality.

## Summary

When delivering continuous ventricular ablation lesions 10 mm is the interlesion distance threshold, below which lesion pairs will remain connected forming more contiguous and effective scars. Additionally, higher contact force should be used in ventricular ablation to create transmural lesions. Lesion volumes as assessed by LGE-MRI accurately correlates with histological specimens. Such imaging techniques help improve practices in the EP lab.

## Data Availability Statement

Data can be made available by authors upon request.

## Ethics Statement

The animal study was reviewed and approved by the Institutional Animal Care and Use Committee at the University of Utah.

## Author Contributions

MM helped fine-tuning the manuscript. CH performed experimental work. CN, YZ, and EK helped with analysis. TA and NM supervised the project. All authors contributed to the article and approved the submitted version.

## Conflict of Interest

NM reports receiving research grant support from Sanofi, Biosense Webster, Boston Scientific, Abbot, and Janssen; provides consulting services to Biosense Webster and Atricure; and received lecture honorarium from BMS, Biotronic, and Sanofi. The remaining authors declare that the research was conducted in the absence of any commercial or financial relationships that could be construed as a potential conflict of interest.

## Publisher’s Note

All claims expressed in this article are solely those of the authors and do not necessarily represent those of their affiliated organizations, or those of the publisher, the editors and the reviewers. Any product that may be evaluated in this article, or claim that may be made by its manufacturer, is not guaranteed or endorsed by the publisher.

## Abbreviations

RFA: Radiofrequency Ablation
PVI: Pulmonary Vein Isolation
AF: Atrial Fibrillation
AI: Ablation Index
EAM: Electroanatomic Mapping
LGE-MRI: Late Gadolinium Enhancement Magnetic Resonance Imaging
EP: Electrophysiology
MVO: Microvascular Obstruction
CF: Contact Force
LV: Left Ventricle
RV: Right Ventricle
LA: Left Atrium.
