# Supplementary material for: Predictors of Lesions Contiguity and Transmurality in Canine Ventricular Models After Catheter Ablation
Source: Front Cardiovasc Med. 2022 Jun 23;9:920539. doi: 10.3389/fcvm.2022.920539 (PMC9260253; doi:10.3389/fcvm.2022.920539)

10.3389/fcvm.2022.920539

Supplementary table: Characteristics of different pairs of lesions.

Pair	EAM_dist (mm)	Connected	Chamber	Duration (s)	Power (W)	Temperature (Celsius)	Force (g)	Acute lesion size (cm^3)	Location
1	7.4	Yes	LV	30	35	35	16	1.83	free wall
			LV	30	35	33	21	1.83	free wall
2	9.78	Yes	LV	30	35	37	19	0.52	free wall
			LV	30	35	30	15	0.52	free wall
3	11.18	Yes	LV	30	35	30	15	1.24	free wall
			LV	30	35	37	19	1.24	free wall
4	12.35	Yes	RV	30	35	33	10	1.12	Septum
			RV	30	35	31	11	1.12	Septum
5	14.36	Yes	RV	30	35	29	13	0.74	free wall
			RV	30	35	35	15	0.74	free wall
6	11.63	Yes	RV	10	50	33	15	0.3	Septum
			RV	10	50	33	15	0.3	Septum
7	13.86	Yes	LV	20	50	27	14	2.61	free wall
			LV	20	50	27	12	2.61	free wall
8	19.01	No	LV	30	35	36	8	0.92	Septum
			RV	30	35	44	10	0.97	Septum
9	11.68	No	RV	30	35	31	29	0.43	free wall
			RV	30	35	34	22	0.7	free wall
10	14.5	No	LV	10	50	39	10	0.18	free wall
			LV	10	50	32	21	0.52	free wall
11	16.43	No	LV	20	50	27	14	1.38	free wall
			LV	20	50	26	15	1.4	free wall

12	14.92	No	LV	20	50	33	8	0.37	free wall
			LV	20	50	33	12	0.51	free wall
13	16.95	No	LV	20	50	35	16	0.28	free wall
			LV	20	50	34	19	0.75	free wall
14	15.09	No	LV	20	50	34	10	0.37	Septum
			LV	20	50	46	17	0.74	Septum
15	21.27	No	RV	20	50	27	8	0.51	Septum
			RV	20	50	26	12	0.71	Septum
16	15.9	No	RV	20	50	N/A	8	0.13	Septum
			RV	20	50	N/A	15	0.25	Septum
17	16.59	No	RV	20	50	N/A	20	0.48	free wall
			RV	20	50	N/A	15	0.5	free wall
18	16.32	No	RV	20	50	32	6	0.18	free wall
			RV	20	50	34	8	0.25	free wall
19	15.4	No	RV	20	50	34	10	0.09	free wall
			RV	20	50	31	11	0.42	free wall

Supplementary Figure 1: Bland Altman Plots showing correlation between histology and LGE-MRI assessment of lesions.

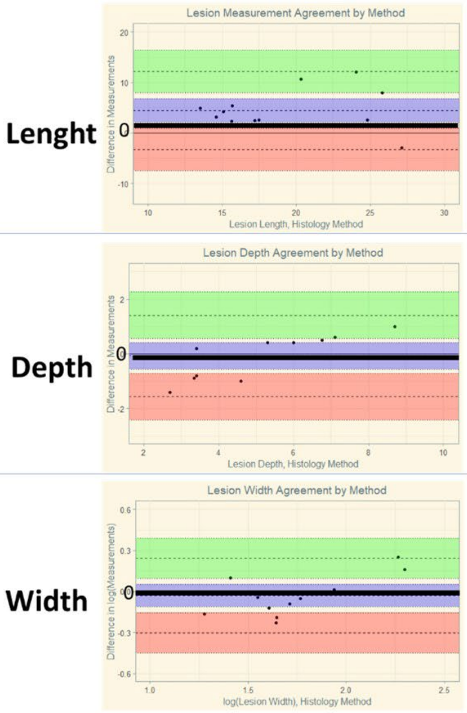

Length	Mean	95% CI (Low)	95% CI (High)
Bias	4.4	2.1	6.8
Upper Level of Agreement	12.2	8.0	16.4
Lower Level of Agreement	-3.3	-7.5	0.9

log(Depth)	Mean	95% CI (Low)	95% CI (High)
Bias	-0.03	-0.11	0.05
Upper Level of Agreement	0.24	0.09	0.38
Lower Level of Agreement	-0.30	-0.45	-0.16

Width	Mean	95% CI (Low)	95% CI (High)
Bias	-0.1	-0.6	0.4
Upper Level of Agreement	1.4	0.6	2.3
Lower Level of Agreement	-1.6	-2.4	-0.7

10.3389/fcvm.2022.920539

Supplementary Figure 2: (A) Difference between averages of the acute and chronic lesion volumes in connected, unconnected, and all lesions; (B) Difference between averages of the acute and chronic lesion volumes using different ablation parameters. n=single lesion; 2n=one pair of two lesions

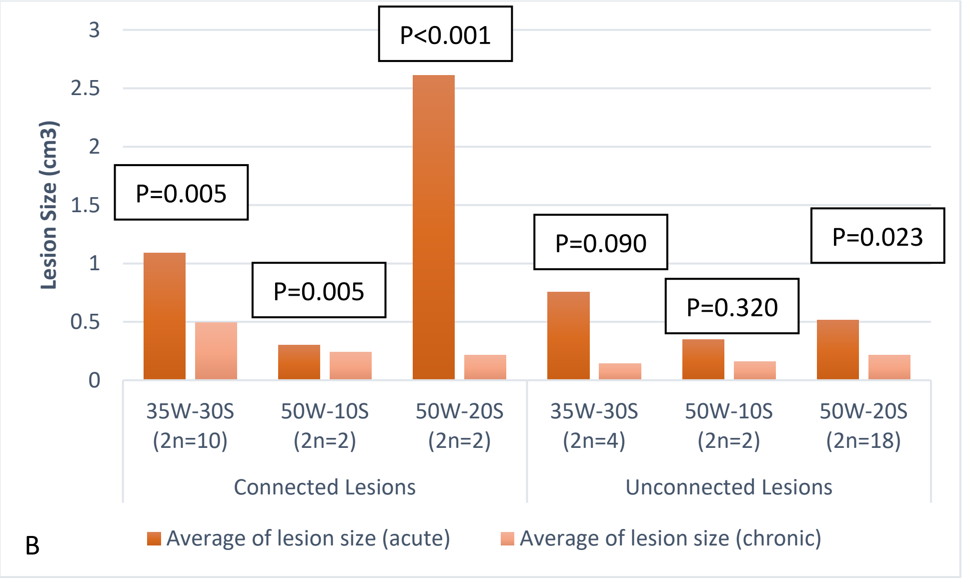

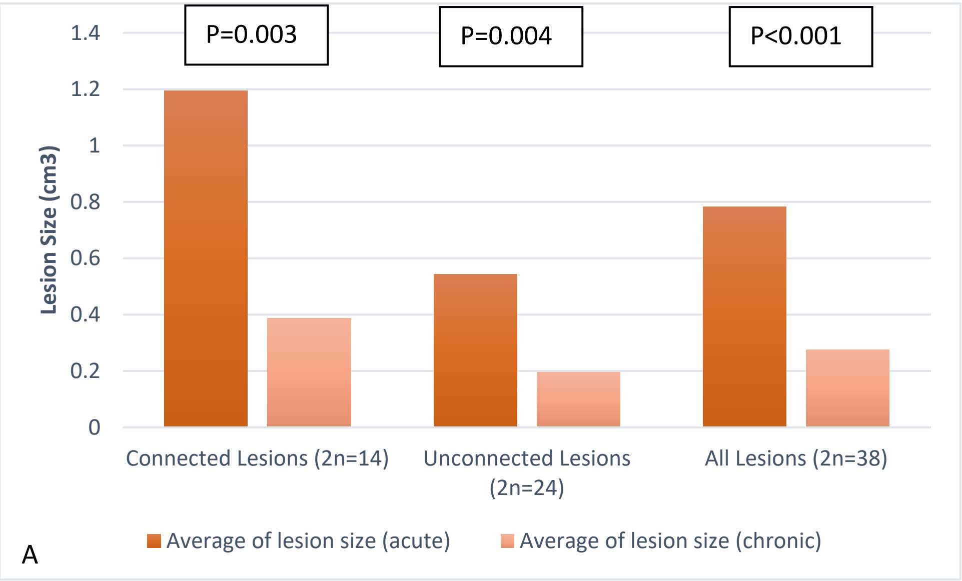

Supplement: Supplementary file 1 [file Data_Sheet_1.pdf]
